# Safety of low-molecular-weight heparin compared to unfractionated heparin in hemodialysis: a systematic review and meta-analysis

**DOI:** 10.1186/s12882-017-0596-4

**Published:** 2017-06-07

**Authors:** Hind Harrak Lazrak, Émilie René, Naoual Elftouh, Martine Leblanc, Jean-Philippe Lafrance

**Affiliations:** 1 Maisonneuve-Rosemont Hospital Research Center, Montreal, Canada; 2 Department of Medicine, University of Montreal, Montreal, Canada; 3 Division of Nephrology, Maisonneuve-Rosemont Hospital, 5415, boul. de l’Assomption, Montreal, QC H1T 2M4 Canada

**Keywords:** Bleeding, Chronic renal dialysis, Low molecular weight heparin, Meta-analysis, Systematic review, Unfractionated heparin

## Abstract

**Background:**

Low molecular weight heparins (LMWH) have been extensively studied and became the treatment of choice for several indications including pulmonary embolism. While their efficacy in hemodialysis is considered similar to unfractionated heparin (UFH), their safety remains controversial mainly due to a risk of bioaccumulation in patients with renal impairment. The aim of this systematic review was to evaluate the safety of LMWH when compared to UFH for extracorporeal circuit (ECC) anticoagulation.

**Methods:**

We used Pubmed, Embase, Cochrane central register of controlled trials, Trip database and NICE to retrieve relevant studies with no language restriction. We looked for controlled experimental trials comparing LMWH to UFH for ECC anticoagulation among end-stage renal disease patients undergoing chronic hemodialysis. Studies were kept if they reported at least one of the following outcomes: bleeding, lipid profile, cardiovascular events, osteoporosis or heparin-induced thrombocytopenia. Two independent reviewers conducted studies selection, quality assessment and data extraction with discrepancies solved by a third reviewer. Relative risk and 95% CI was calculated for dichotomous outcomes and mean weighted difference (MWD) with 95% CI was used to pool continuous variables.

**Results:**

Seventeen studies were selected as part of the systematic. The relative risk for total bleeding was 0.76 (95% CI 0.26–2.22). The WMD calculated for total cholesterol was −28.70 mg/dl (95% CI -51.43 to −5.98), a WMD for triglycerides of −55.57 mg/dl (95% CI -94.49 to −16.66) was estimated, and finally LDL-cholesterol had a WMD of −14.88 mg/dl (95% CI -36.27 to 6.51).

**Conclusions:**

LMWH showed to be at least as safe as UFH for ECC anticoagulation in chronic hemodialysis. The limited number of studies reporting on osteoporosis and HIT does not allow any conclusion for these outcomes. Larger studies are needed to evaluate properly the safety of LMWH in chronic hemodialysis.

**Electronic supplementary material:**

The online version of this article (doi:10.1186/s12882-017-0596-4) contains supplementary material, which is available to authorized users.

## Background

Low molecular weight heparins (LMWH) are among the preferred anticoagulant for preventing and treating venous thrombosis [1]. A meta-analysis of randomized controlled trials showed similar efficacy between LMWH and unfractionated heparin (UFH) for acute deep venous thrombosis prevention, and no bleeding risk difference [2] with a reduced mortality rate in favor of LMWH. [2] LMWH are replacing UFH as the first line treatment for pulmonary embolism and unstable angina, a choice mainly due to their predictable effect and convenient use [3].

Despite the fact that the European Best Practice Guidelines for hemodialysis recommended the use of LWMH for the extracorporeal circuit (ECC) anticoagulation, UFH remains the most frequent choice for hemodialysis treatment in North America [4]. While cost may be the main argument for not using LMWH in hemodialysis, their safety remains a major concern. UFH is metabolized by both hepatic and renal pathways but LMWH are mainly cleared through the kidneys leading to a potential bioaccumulation and an increased risk of hemorrhage [5]. A meta-analysis conducted by Lim et al. evaluated the safety and efficacy of LMWH in hemodialysis, finding no difference between LMWH and UFH for both bleeding and thrombosis of the ECC. While the sample was large enough to evaluate efficacy (thrombosis), limited data was available to evaluate the risk of bleeding, leading to large confidence intervals (CI), and therefore limited conclusions [6].

UFH is known to modify the lipid profile, to induce osteoporosis, and to carry a risk of heparin-induced thrombocytopenia (HIT). However, the impact of LMWH on these outcomes in hemodialysis remains unclear [7].

The aim of this systematic review was to evaluate the safety of LMWH compared to UFH as an anticoagulant of the ECC among patients undergoing chronic hemodialysis. The primary outcomes were the risk of minor and major bleedings, cardiovascular events and osteoporotic fractures. Secondary outcomes were changes in lipid profile, osteoporosis and HIT.

## Methods

Two independent reviewers (HHL and ER) conducted the study selection, validity assessment and data extraction with disagreement solved by a third reviewer (JPL).

### Performed searches

Databases were screened to retrieve prospective experimental studies comparing LMWH to UFH for anticoagulation of the ECC during hemodialysis for patients with end-stage renal disease (ESRD). The search strategy was developed with a professional librarian. We searched Pubmed (from start up to January 2016), Embase (1974 to 2016 week 1), Cochrane central register of controlled trials (from start to January 2016), Trip database, the National Institute for Health and Care Excellence (NICE) and clinicaltrials.gov/ with no language restriction. Performed searches were conducted by one reviewer (HHL). Reference lists of selected studies were screened manually and authors were contacted when additional data was needed (HHL and ER) Additional file 1.

### Study selection

Studies were selected if they fulfilled the following criteria: a) Patients were adults with ESRD undergoing chronic hemodialysis (incident or prevalent); b) LMWH was compared to UFH for anticoagulation of the ECC during hemodialysis; c) at least one of the outcomes of interest was reported (minor or major bleeding, lipid profile changes for total cholesterol, LDL-cholesterol, triglycerides, cardiovascular events, osteoporosis, osteoporotic fractures, HIT); d) the design was a prospective randomized or non-randomized cross-over or a parallel randomized study. A study was excluded if: a) it was a non-randomized parallel-design; b) it was using historical data; or c) the results were published more than once in which case only the most complete study was kept.

### Quality assessment

Quality of each study was assessed using the Cochrane risk of bias tool [8] for randomized trials or the risk of bias in non-randomized studies (Robins-I) [9]. The Cochrane risk of bias tool evaluates the methodology of the study and the potential biases in the research question context. [8] There is no final score but results are reported as low, unclear or high risk of bias for different potential biases. The Robins-I uses a similar approach but is adapted to non-randomized trials.

### Data extraction

A common form was used to ascertain extraction of the complete study characteristics and outcomes of interest as follows: a) bleeding events classified as major and minor when specified by the authors; b) cardiovascular events; c) LDL, total-cholesterol and triglycerides levels at start and end of study for both treatment and control; d) osteoporotic fractures; e) osteoporosis diagnostic; f) bone density changes; g) OPG/RANKL biomarkers affecting bone density; and h) HIT.

### Statistical analysis

The weighted kappa statistics was used to assess between reviewers agreement for study selection and quality assessment [10]. We calculated relative risks (RR) and 95% confidence intervals (CI) for dichotomous clinical data. A pooled weighted mean difference (WMD) and its 95% CI were used to compare continuous outcomes. The pooled overall effect for both RR and WMD was estimated using the method described by DerSimonian and Laird [11] with a random effect model for heterogeneous data. Heterogeneity was evaluated using the I^2^ statistic using a 60% significance threshold. Publication bias was assessed visually using a funnel plot and using the Egger test [12]. All analyses were performed with Stata IC 11.0.

### Sensitivity analyses

The pooled WMD and 95% CI were calculated based on the pre- and post-treatment measures using their standard deviations to derive the WMD’s standard deviation [8]. In the main analysis, the correlation factor was fixed at 0.5 based on the assumption that repeated measures would be moderately correlated. We tested the robustness of this assumption with correlation factors of 0.3 and 0.8 as sensitivity analyses.

## Results

### Identification and study selection

**Fig. 1:**
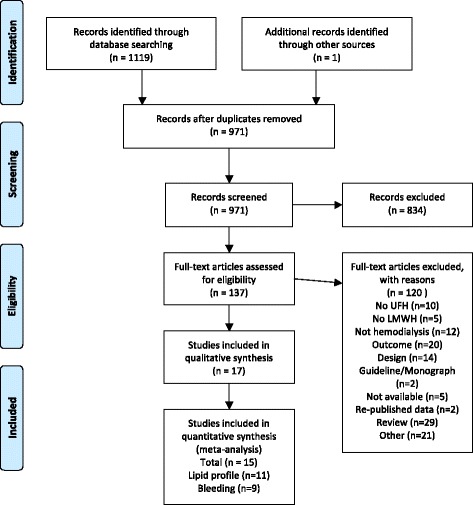
Study selection flow chart adapted from the PRISMA flowchart model. *osteoporosis was not pooled with a meta-analysis (2 studies)

From a total of 971, we identified 17 studies [13–28] that met our pre-defined selection criteria (Fig. 1). The inter-rater agreement was excellent with a weighted kappa of 0.94 for study selection. In the meta-analysis, we included nine studies [13–15, 22–24, 26–28] that recorded bleeding episodes and 11 studies [13, 17–21, 23, 24, 26–28] evaluating lipid changes. Of note, when a study evaluated the same outcome within two different groups of patients and different settings we considered each part of the article as an independent study for both the systematic review and the meta-analysis [17, 28]. All the non-English articles were translated but did not meet criteria. Five studies were with a randomized cross-over design [16, 22, 24, 27, 29], nine were non-randomized cross-over [13–15, 17, 19–21, 25, 28] and three parallel design with randomization [18, 23, 26]. Two studies were excluded from the review since they reported the same results as studies included in the present review. Sabri et al. [30] was excluded for presenting the same data and results as Al-Saran et al. [13]. The same situation occurred between Schneider el al [31] and Schmitt el al [25].

### Characteristics of studies and patients

Characteristics of studies are listed in Table 1. The number of patients ranged from five to 110, and follow-up ranged between one and 60 months. Various types of LMWH were used for the experimental arm, including nadroparin, enoxaparin, tinzaparin, dalteparin, certoparin and fraxiparin. Both men and women were included with age ranging from 15 to 85 years. Exclusion criteria differed between studies but bleeding disorder was a recurrent criterion. Patient characteristics are detailed in Table 2. The most common dialysis schedule was a session of four hours thrice weekly. Hemodiafiltration procedure was also used in three studies [15, 26, 27].

**Table 1:** Characteristics of studies included in the systematic review

Study	Year	Country	Follow-up	Patients enrolled (n) LMWH/UFH (n/n)	Drop-outs (n) LMWH/UFH (n/n)	LMWH type	LMWH mean dose	UFH mean dose	HD/HDF
Cross-over randomized design									
Cianciolo et al. [16]	2011	Italy	2 months	40	0	Nadroparin	64 IU/kg	bolus 50 IU/kg and 30 IU/kg/h	HD
Klejna et al. [29]	2014	Poland	6 months	21	0	Enoxaparin	5280 IU ± 1120 IU	4881 IU ± 1916.3 IU	HD
Lord et al. [22]	2002	Canada	2 months	32	2	Tinzaparin	4318.2 IU ± 702.8 IU	8207.9 IU ± 2530.9 IU	HD
Saltissi et al. [24]	1999	Australia	6 months	36	5	Enoxaparin	100 IU/kg	bolus 50 IU/kg and 1000 IU/h	HD
Stefoni et al. [27]	2002	Italy	36 months	54	7	Nadroparin	64 IU/kg	bolus 1500 IU and 1500 IU and 1500 IU ± 500 IU perfusion	HDF
Cross-over non-randomized design									
Al-Saran et al. [13]	2010	Saudi Arabia	12 months	23	0	Tinzaparin	2000–3000 IU	5000 IU	HD
Bambauer et al. [14]	1990	Germany	12 months	27	6	Dalteparin	4216 IU ± 2237 IU	4958 IU ± 2561 IU	HD
Bramham et al. [15]	2008	United Kingdom	4 months	110	2	Tinzaparin	2500–5000 IU	1000 IU ± 500–2000 IU	HDF
Deuber et al. (part 1) [17]	1991	Germany	60 months	5	0	Non specified	Non specified	29–143 IU/kg	HD
Deuber et al. (part 2) [17]	1991	Germany	24 months	5	0	Non specified	Non specified	29–143 IU/kg	HD
Kronenberg et al. [19]	1995	Austria	12 months	24	0	Certoparin	44.7 IU/kg ± 21.5 IU/kg	25.1 IU/kg ± 7.8 IU/kg	HD
Lai et al. [20]	2001	China	24 months	40	6	Nadroparin	4100–6150 IU	5000–7000 IU	HD
Leu et al. [21]	1998	Taiwan	4 months	20	0	Dalteparin	2413 IU ± 954 IU	2413 IU ± 954 IU	HD
Schmitt et al. [25]	1993	Germany	12 months	22	0	Dalteparin	bolus 1500 IU and 675 IU/h ± 284 IU/h	bolus 1500 IU and 1031 IU/h ± 342 IU/h	HD
Yang et al. (part 1) [28]	1998	Taiwan	1 month	10	0	Fraxiparin	10,000 ICU	bolus 1800 IU ± 675 IU and 895 IU/h ± 340 IU/h	HD
Yang et al. (part 2) [28]	1998	Taiwan	12 months	10	2	Fraxiparin	192.9 ICU/kg ± 3.8 ICU/kg	bolus 1800 IU ± 675 IU and 895 IU/h ± 340 IU/h	HD
Randomized parallel design									
Elisaf et al. [18]	1997	Greece	12 months	76 38/38	0	Tinzaparin	3000 IU	5000–7000 IU	HD
Nurmohamed et al. [23]	1991	The Netherlands	6 months	70 35/35	13 8/5	Nadroparin	150–200 IU/kg	bolus 2500 IU and 600–2200 IU/h	HD
Schrader et al. [26]	1988	Germany	12 months	70 35/35	8 5/3	Dalteparin	bolus 36.8 IU/kg ± 17.3 IU/kg and 12.2 IU/kg/h ± 5.0 IU/kg/h	bolus 58.3 IU/kg ± 26.3 IU/kg and 16.6 IU/kg/h ± 6.7 IU/kg/h	HDF

*HD* Hemodialysis, *HDF* Hemodiafiltration

**Table 2:** Participants characteristics

Study	Mean age + SD (years)	Age range (years)	Male/Female (n/n)	Dialysis duration and frequency	Inclusion criteria	Exclusion criteria
Cross-over with randomization						
Cianciolo et al.	63.3 ± 7.2	42–72	21/19	4 h 3×/wk	chronic HD, age 18+, stable, AVF	gastrointestinal bleeding, acute cardiovascular event 3 months before, malignancy, coagulation disorders, DVT, immunosupressive therapy, acute vasculitis, liver disease, active infection, diabetes, enrolled in other clinical trial
Klejna et al.	68.2	44–82	11/10	4–5 h 3×/wk	Chronic HD	HIV, Hepatitis B, Hepatitis C, VTE, gastrointestinal bleeding, coagulation disorders
Lord et al.	66.6 ± 14.8	NS	17/15	4 h 3×/wk	Chronic HD	Patients with catheters, with bleeding diathesis in last 3 months, with thrombocytopenia, hepatic failure, oral anticoagulation (but not antiplatelets)
Saltissi et al.	NS	22–85	17/19	3–5 h 3×/wk	Chronic HD	bleeding disorders, anticoagulation therapy (warfarin, aspirin)
Stefoni et al.	63.7 ± 7	NS	39/15	4 h 3×/wk	Chronic HD for at least 12 months	active gastrointestinal bleeding, myeloproliferative disorders, malignant diseases, hereditary deficiency of coagulation factors, LAC phenomenon, antiphospholipid syndrome
Cross-over without randomization						
Al-Saran et al.	46.83 ± 14.63	NS	17/6	3–4 h 3×/wk	at least 6 months on HD prior to study	bleeding disorders, anemia with hemoglobin levels less than 10 g/dL, recent trauma, surgery, infectious disease or hemorrhagic disorder (< 1 month) in addition to those receiving oral or other forms of anticoagulant therapy (e.g. warfarin, aspirin), or drugs that could affect heparin activity (e.g. tetracyclines, digitalis, and antihistamines)
Bambauer et al.	60	NS	12/15	NS	Chronic HD	NS
Bramham et al.	61 ± 15	NS	65/45	3–4 h 3×/wk	Chonic HD on monitoring shift	Renal transplant, transferred to satellite unit, switched to PD, on warfarin
Deuber et al. (part 1)	53 ± 7	47–65	NS	4 h 3×/wk	chronic HD for at least 18 months	NS
Deuber et al. (part 2)	50 ± 18	20–67	NS	4 h 3×/wk	chronic HD for at least 18 months	NS
Kronenberg et al.	44.7 ± 16.8	NS	13/11	3.5–5 h 3×/wk	in pre-dialysis	diabetes, bleeding disorders, oral anticoagulants, lipid lowering drugs
Lai et al.	42.2 ± 5.2	24–60	25/15	10–16 h/wk	Chronic HD	diabetes, primary hyperlipidemia
Leu et al.	57.8 ± 9.8	NS	7/13	4 h 3×/wk	Chronic HD at least 6 months	pts with lipid lowering drugs except non-diabetic pts. under lovastatin for >6 months, known hemorrhagic diathesis, low platelet count, liver insufficiency, hypersensitivity to heparin
Schmitt et al.	58.6	37–72	13/9	4–5.8 h	chronic HD, cholesterol >200 mg/dL	diabetes, concomittant drug treatment (lipid lowering drugs, COX inhibitors)
Yang et al. (part 1)	44 ± 15	NS	7/3	3×/wk	chronic HD, non diabetic	NS
Yang et al. (part 2)	57 ± 6.4	NS	7/3	3×/wk	chronic HD with diabetes type II	NS
Parallel with randomization						
Elisaf et al.	NS	15–61	NS	4 h 3×/wk	chronic HD	diabetes, hyperlipidemia (primary or secondary)
Nurmohamed et al.	NS	NS	NS	4–6 h 2-3×/wk	chronic HD	NS
Schrader et al.	54.0 ± 15.2 (LMWH) 51.6 ± 17.9 (UFH)	NS	21/14 (LMWH) 19/16 (UFH)	NS	pre-HD requiring HD, not on heparin in prior 3 months	bleeding disorders, needed antiplatelets or anticoagulants

*NS* not specified, *HD* hemodialysis, *PD* peritoneal dialysis, *hrs* hours, *wk.* week, *DVT* deep venous thrombosis, *VTE* venous thromboembolism, *AVF* arteriovenous fistula

### Quality assessment

**Fig. 2:**
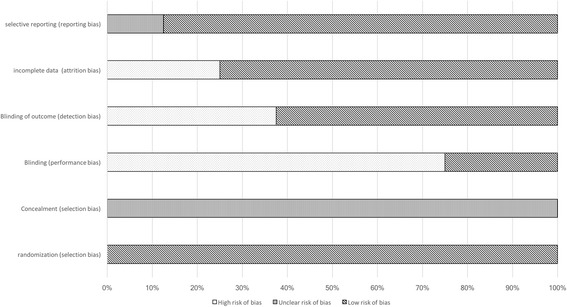
Cochrane risk of bias tool diagram

**Fig. 3:**
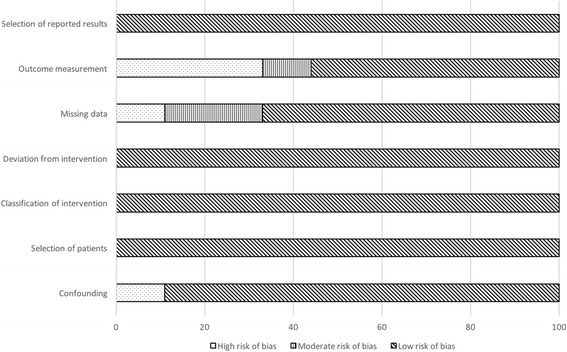
Risk of Bias in non-randomized studies (Robins-I) diagram

Based on the analysis with the Cochrane risk of bias tool for randomized studies (Fig. 2), we observed a high rate of potential performance bias mainly caused by a lack of blinding and only few measures to ensure similar care to the patients independently from heparin type. Blinding, or lack of it, could also affect outcome detection (detection bias). We judged that there was a high risk of bias when the outcome of interest was subjective. Bleeding events were categorized as being minor or major in eight out of nine studies, but most of the time no clear definition was provided except for one study. [27] The lack of definition made the evaluation of bleeding events subjective. In randomized trials, concealment was not specified, leading to an imprecise risk of selection bias for these studies. Overall, attrition and reporting biases were adequately handled through the trials (Fig. 2). Similarly, non-randomized cross-over studies had a moderate to high risk of outcome measurement when bleeding events evaluation was based on subjective assessment due to lack of clear outcome definition. The Robins-I tool points also to a missing data problematic due to drop-outs. (Fig. 3) The weighted kappa was 0.72 for Cochrane risk of bias tool and 0.69 for Robins-I, indicating a substantial agreement between raters.

### Synthesis of individual studies results

Not all studies had a predefined definition of the outcome of interest. Bleeding outcome was specified in the method section in only half of the studies [15, 22, 24, 27, 28]. All except one [14] reported bleeding events as minor or major. Only Stefoni et al. provided a clear definition of major (melena, hematemesis and arterial epistaxis) and minor bleeding (venous epistaxis, subconjuctival hemorrhage or prolonged bleeding from the cannulation site after removal of the dialysis needle) [27]. For Bramham et al., a bleeding event was defined as a prolonged compression time or any other bleeding episode reported during or between dialysis sessions by the patients, including epistaxis or conjunctival bleeding [15]. Among all bleeding events, only two were categorized by the authors as major, and both occurred in the LMWH group. When combining both major and minor bleedings, 24 (6.6%) events were recorded for LMWH compared to 31 (8.5%) for UFH. Bleeding outcomes are presented in Table 3.

**Table 3:** Bleeding events and relative risk with 95% CI for LMWH compared to UFH

Study, year	Total bleeding events LMWH (n/N)	Total bleeding events UFH (n/N)	RR (95% CI)	Comments
Al-Saran et al.	3/23	0/23	7.00 (0.38–128.33)	3 minor bleedings with LMWH, controlled with dose adjustment
Bambauer et al.	3/27	6/27	0.50 (0.14–1.80)	No indication if minor or major bleeding events
Bramham et al.	0/110	4/110	0.11 (0.01–2.04)	4 minor bleeding episodes with UFH
Lord et al.	3/32	8/32	0.38 (0.11–1.29)	LMWH: 1 major and 2 minor bleedings; UFH: 8 minor bleedings
Nurmohamed et al.	3/35	0/35	7.00 (0.37–130.69)	3 minor bleeding events with LMWH
Saltissi et al.^a^	12/36	6/36	2.00 (0.84–4.75)	LMWH:1 major and 11 minor bleedings; UFH: 6 minor bleedings
Stefoni et al.	0/54	7/54	0.07 (0.00–1.14)	7 minor bleeding events with UFH
Schrader et al.^b^	0/35	0/35	excluded	n/a
Yang et al. (part 1)^b^	0/10	0/10	excluded	n/a
Summary	24/362	31/362	0.76 (0.26–2.22)	

^a^Details of bleeding events extracted from Lim et al.

^b^Studies excluded due to “zero cells” in both LMWH and UFH groups

Cardiovascular events were reported in two studies; while Bambauer et al. [14] recorded two events with LMWH and three for UFH, Schrader et al. [26] observed two events with LMWH and one event with UFH. The majority of the studies showed a decrease in lipid levels using LMWH except for three which reported either an increase or no changes for cholesterol, [19, 26] LDL-cholesterol [19, 24] and triglycerides [24, 26]. The beneficial effect on lipids was less pronounced when using UFH where cholesterol, LDL-cholesterol and triglycerides increased or remained stable in five, [17, 21, 24–26] three [21, 24, 25] and six [17, 21, 25–28] studies, respectively (Tables 4-S1-S2).

**Table 4:** LDL-cholesterol weighted mean difference

Study	LMWH	UFH	
	mean change, SD (mg/dl)	mean change, SD (mg/dl)	WMD (95% CI) (mg/dl)
Al-Saran et al.^a^	−30.94 ± 39.52	−20.11 ± 44.69	−10.83 (−35.21, 13.55)
Elisaf et al.	−18 ± 36.86	−2 ± 32.78	−16.00 (−31.69, −0.31)
Kronenberg et al.	12.3 ± 36.68	−16.5 ± 33.84	28.80 (8.83, 48.77)
Lai et al.^a^	−2.70 ± 36.93	−6.57 ± 45.82	3.87 (−14.37, 22.11)
Leu et al.^a^	−35.96 ± 35.03	31.71 ± 36.80	−67.67 (−89.94, −45.40)
Saltissi et al.^a^	0.39 ± 29.01	1.55 ± 30.55	−1.16 (−14.92, 12.60)
Schmitt et al.	−27 ± 51.10	21 ± 48.12	−48.00 (−77.33, −18.67)
Summary			−14.88 (−36.27, 6.51)

^a^Results were expressed in mmol/L, we converted them in mg/dl to be able to pool them

No study evaluated the incidence of osteoporotic fractures. Osteoprotegerin (OPG) and receptor activator of nuclear factor-κB ligand (RANKL) levels were measured in two studies. The OPG/RANKL system is involved in bone metabolism. No significant difference between LMWH and UFH (Additional file 2: Table S3) was found [16, 29]. Lai et al. [20] measured the bone densitometry with dual energy X-ray and observed an increase of bone mass density (BMD) in ward’s triangle by 0.75% after switching to LMWH. This increase did not reach statistical significance (*p* = 0.11). The mean BMD in the same region decreased by 2.38% after patients were back on UFH [20]. They did not observe a similar trend for the other sites investigated.

Bramham et al. [15] reported that they did not observe any case of thrombocytopenia among their patients during the study follow-up for both LMWH and UFH.

### Meta-analysis results

**Fig. 4:**
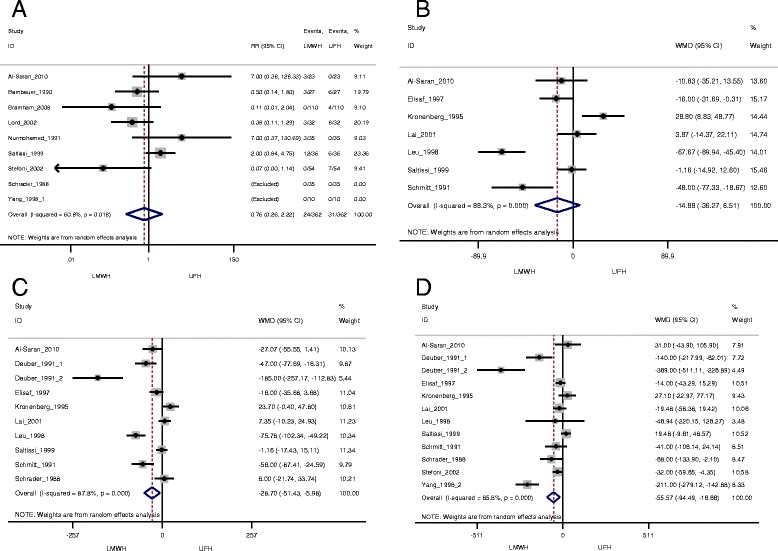
Meta-analysis results for (**a**) total bleeding relative risk and confidence interval, (**b**) LDL-cholesterol, (**c**) Total cholesterol and (**d**) Triglycerides weighted mean difference and confidence interval for LMWH compared to UFH. Abbreviations: WMD, weighted mean difference, CI, confidence interval

Schrader et al. [26] and Yang et al. [28] were excluded from the analysis since they did not have any bleeding event in both LMWH and UFH groups. The overall RR for total bleeding (minor and major) with LMWH compared to UFH was 0.76 (95% CI: 0.26, 2.22) using a random effect model. The I^2^ statistic for heterogeneity was statistically significant (*p* = 0.018) (Fig. 4a). Compared to UFH, LMWH showed lower cholesterol level, with a pooled WMD of −28.70 mg/dl (95% CI: -51.43, −5.98) (Fig. 4c). A pooled WMD of −55.57 mg/dl (95% CI: -94.49, −16.66) was calculated for triglycerides showing lower levels in the LMWH group compared to UFH (Fig. 4d). A similar trend was observed with LDL-cholesterolbut the results were not statistically significant (pooled WMD: −14.88 mg/dl (95% CI: -36.27, 6.51)) (Fig. 4b). Results were pooled using random effect model as the heterogeneity test was statistically significant. Because of the small number of studies, visual interpretation of the funnel plots to assess possible publication bias was inconclusive (Additional file 2). The Egger test results indicated a potential publication bias (data not presented).

### Sensitivity analyses

Using different correlation factors to calculate the standard deviation of the mean changes for cholesterol, LDL-cholesterol and triglycerides rendered similar results. With a correlation factor of 0.3 the pooled WMD for cholesterol, triglycerides and LDL were, −27.38 mg/dl (95% CI: -50.78, −3.98), −53.76 mg/dl (95% CI: -94.98, −12.53) and −14.49 mg/dl (95% CI: -35.73, 6.75) respectively. A correlation factor of 0.8 resulted in pooled WMD for cholesterol, triglycerides and LDL of −32.25 mg/dl (95% CI: -55.08, −9.42), −60.78 mg/dl (95% CI: -97.74, −23.83) and −15.49 mg/dl (95% CI: -37.56, 6.57) respectively.

## Discussion

This systematic review reports on safety of LMWH compared to UFH when used for anticoagulation of the extracorporeal circuit during hemodialysis. There was no statistical difference for risk of bleeding in the LMWH compared to the UFH group. We also showed lower levels of total cholesterol and triglycerides when exposed to LMWH compared to UFH. LDL-cholesterol levels tended to be lower using LMWH but the difference did not reach statistical significance. Because of the small numbers of studies, we were not able to estimate the differential risk of cardiovascular events, osteoporotic fractures and HIT.

A decade after the publication of the first meta-analysis published by Lim et al. [6], we conducted a new systematic review to re-evaluate LMWH bleeding risk in hemodialysis compared to UFH by including all new relevant studies. Lim et al. found similar overall bleeding risk (RR = 0.96, 95% CI: 0.27, 3.43), a result that is consistent with our study. Our updated meta-analysis adds to this earlier study by narrowing the CI and reducing the upper limit from three to two, supporting that, at least, LMWH does not carry a higher risk of bleeding. Our results are also consistent with observed bleeding risk among patients without renal failure using LMWH for their primary indication [32, 33]. Renal-dependent clearance of LMWH remains a concern. A prospective observational cohort study evaluated the bioaccumulation of dalteparin when administered at the therapeutic dose twice a day. Patients with a glomerular filtration rate (GFR) inferior to 30 mL/min/1.73 m^2^ showed a significant bioaccumulation of dalteparin at the end of the study [34]. On the other hand, two studies were conducted among critically ill patients with severe renal insufficiency, and did not observe bioaccumulation of dalteparin using a prophylactic dose of 5000 IU once a day [35, 36]. The later schedule is closer to the hemodialysis context.

No prior systematic review compared LMWH and UFH for cardiovascular events and lipid profile in hemodialysis. UFH is known to activate lipoprotein lipase and hepatic lipase and therefore to affect lipid profil [7]. Unfortunately, data on cardiovascular events were limited. Although it was demonstrated in the SHARP study [37] and a meta-analysis [38] that, for patients with chronic kidney disease, lowering LDL-cholesterol by 38.61 mg/dl (1 mmol/L) induced a reduction of 17% of major atherosclerotic events, in dialysis-dependent patients there is only a weak association between LDL-cholesterol level and the risk of cardiovascular event [39]. Indeed, low LDL-cholesterol level could be associated with a high risk of cardiovascular event in dialysis patients when there is coexistence of malnutrition and inflammation [40, 41]. While our results were in favor of LMWH, it remains uncertain whether the decrease would translate into lower cardiovascular events among ESRD patients.

Our meta-analysis is the first to compare LMWH and UFH for bone metabolism in hemodialysis. The exact mechanism involved in osteoporosis induced by UFH remains unclear. One hypothesis suggests a suppression of osteoblast formation and activation of osteoclasts promoting bone loss [5]. Unfortunately, no study compared osteoporotic fractures. Different studies presented the OPG/RANKL system as an actor of bone remodeling [42, 43]. No difference was detected in the studies retrieved [16, 29]. While it is reported that LMWH cause less bone loss among pregnant women, these results were not replicated among hemodialysis patients [7]. Uncertainty was raised in the Kidney Disease Improving Global Outcomes (KDIGO) clinical guidelines in 2009 about the reliability of BMD measured with dual energy X-ray [44]. However a systematic review and meta-analysis conducted by Bucur et al. [45] showed that BMD is lower in chronic kidney disease (CKD) patients with fracture compared to CKD patients without fracture, suggesting that BMD could still be clinically relevant to evaluate fracture risk in CKD.

Thrombocytopenia is a rare but severe adverse reaction to heparin. Although Bramham et al. [15] did not observe any HIT; we cannot reach any conclusion regarding this outcome. The incidence of thrombocytopenia was reported between 1 and 3% with UFH, while it was less than 1% with LMWH [46]. UFH binds to the platelet factor 4 (PF4). Antibodies then recognize this complex, which leads to an autoimmune reaction and thrombocytopenia [47]. Because a long chain of saccharides is needed to bind to PF4, and that LMWH are composed of short chains, it is believed that LMWH is less likely to form such complexes [48].

Our review has some limitations. Except for bleeding and cardiovascular events, we could not obtain data on every one of our primary outcomes. Our analyses were based on the secondary outcomes, and they might not be effective surrogates to infer on the main outcomes. The bleeding risk with LMWH may have been misestimated as some of the studies included an exploratory phase to determine the right LMWH dosage, which is partly based on occurrence of bleeding events, while UFH treatment arm did not go through such phase since patients were already stable under that regimen.

The potential publication bias detected must be taken into consideration when interpreting the results. Although the use of objective searching methods we could not eliminate this bias. Because of the lack of clear and consistent definition of major and minor bleeding in the retrieved studies, we opted to evaluate the risk of total bleeding. However, it would have been interesting to measure the risk of hemorrhage according to its severity, where major bleeding has a greater impact on the patient but also on the type of care needed [49]. A standardized definition of major and minor bleeding needs to be used in future investigations; such definition is already provided by the subcommittee on control of anticoagulation of the scientific and standardization committee of the international society on thrombosis and haemostasis [49].

The random effect model cannot compensate for the entire heterogeneity found between studies. High heterogeneity could be caused by multiple factors. The inclusion of different study designs in our review. We believe that the crossover and parallel-design should not be different for the measured outcomes in this context. Because of the small number of trials, it was not possible to analyze LMWH separately. LMWH have different pharmacokinetics and kidney failure does not alter similarly their elimination. For instance, enoxaparin might seem safe when there is clear evidence of its bioaccumulation [50, 51]. Finally, the trials used different doses and had variable follow-up time. The dosage, the use of low or high flux dialysis, types of membranes, hemodiafiltration and hemodialysis procedures, all these factors would contribute to the measured heterogeneity.

## Conclusion

In conclusion, the available data does not allow to determine which heparin form is safer when used for anticoagulation of the extracorporeal circuit during hemodialysis. The risk of hemorrhage was not statistically significant when LMWH was compared to UFH, and lipid levels remained comparable or lower with LMWH. As for osteoporosis and thrombocytopenia, the data does not allow making a reliable comparison with UFH. The potential sources of bias discussed earlier and the quality level of the retrieved studies hinder the interpretation of observed results. LMWH have been used in hemodialysis for many years, but we are still in dire need of trials addressing the aforementioned limitations. Future studies need to use larger sample size during a sufficiently long follow-up and a clearer definition of the outcome measured to make their results usable and interpretable. Anticoagulation is mandatory for most patients undergoing hemodialysis treatments, and the choice of anticoagulation agent must be supported by stronger evidence.

## Additional files

Additional file 1: Literature search strategies for Pubmed, Embase and Cochrane as used in this study are detailed in this document. (PDF 16 kb)

Additional file 2: Supplementary material providing the meta-analysis results for cholesterol (Table S1.) and triglycerides (Tables S2.), OPG/RANKL ratios (Table S3.), sensitivity analysis results (Table S4.) and funnel plots for publication bias. (PDF 177 kb)

## Abbreviations

BMD: Bone mass density
CI: Confidence intervals
CKD: Chronic kidney disease
ECC: Extracorporeal circuit
ESRD: End-stage renal disease
HIT: Heparin-induced thrombocytopenia
KDIGO: Kidney Disease Improving Global Outcomes
LMWH: Low molecular weight heparins
MWD: Mean weighted difference
PF4: Platelet factor 4
RR: Relative risks
UFH: Unfractionated heparin
WMD: Weighted mean difference
