# Supplementary material for: Safety of low-molecular-weight heparin compared to unfractionated heparin in hemodialysis: a systematic review and meta-analysis
Source: BMC Nephrol. 2017 Jun 7;18:187. doi: 10.1186/s12882-017-0596-4 (PMC5463373; doi:10.1186/s12882-017-0596-4)
Supplement: Supplementary file 1 — Literature search strategies for Pubmed, Embase and Cochrane as used in this study are detailed in this document. (PDF 16 kb) [file 12882_2017_596_MOESM1_ESM.pdf]

Literature search strategy for Pubmed:

#1AddSearch "Heparin, Low-Molecular-Weight"[Mesh:NoExp] OR LMWH OR "Low Molecular Weight Heparin" OR "Low-Molecular-Weight Heparin"

#2AddSearch Dalteparin[Mesh] OR Tedelparin OR "FR-860" OR "FR 860" OR "FR860" OR "Kabi-2165" OR "Kabi 2165" OR "Kabi2165" OR "Dalteparin Sodium" OR Fragmin OR "Pfizer Brand of Dalteparin Sodium" OR Fragmine OR Dalteparine OR "Dalteparine sodique" OR "Dalteparin sodique" OR "Dalteparine sodium" OR "Dalteparin natrium" OR Ligofragmin OR "Ligo fragmin"

#3AddSearch Enoxaparin[Mesh] OR Enoxaparine OR Enoxaparin OR "Enoxaparin sodium" OR "Enoxaparine sodium" OR "Enoxaparin sodique" OR "Enoxaparine sodique" OR "PK-10,169" OR "PK 10,169" OR "PK10,169" OR "PK-10169" OR "PK 10169" OR "PK10169" OR "EMT-967" OR "EMT 967" OR "EMT967" OR "Lovenox" OR "Clexane" OR "EMT-966" OR "EMT 966" OR "EMT966" OR "Klexane"

#4AddSearch Nadroparin[Mesh] OR Nadroparine OR Nadroparin OR "Nadroparin Calcium" OR "Nadroparine Calcium" OR "Nadroparin Calcique" OR "Nadroparine Calcique" OR Fraxiparin OR Fraxiparine OR "CY 216" OR "CY-216" OR "CY216" OR "LMF CY-216" OR "LMF CY 216" OR "LMF CY216" OR "Fraxiparina"

#5AddSearch tinzaparin [Supplementary Concept] OR tinzaparin sodium OR "tinzaparine sodium" OR "tinzaparin sodique" OR "tinzaparine sodique" OR tinzaparin OR tinzaparine OR "Innohep" OR "Leo brand of tinzaparin sodium" OR "Bristol-Myers Squibb brand of tinzaparin sodium" OR Logiparin

#6AddSearch reviparin [Supplementary Concept] OR reviparine OR reviparin OR "reviparine-sodium" OR "reviparine sodium" OR "reviparin-sodium" OR "reviparin sodium" OR "reviparine sodique" OR "reviparin sodique" OR "LU 47311" OR "LU-47311" OR "Clivarin" OR "Abbott brand of reviparin-sodium" OR "Clivarine" OR "ICN brand of reviparin-sodium"

#7AddSearch bemiparin [Supplementary Concept] OR "Bemiparin Sodium" OR "Bemiparine sodium" OR "Bemiparin sodique" OR "Bemiparine sodique" OR Bemiparin OR Bemiparine OR Ibor OR Zivor

#8AddSearch #1 OR #2 OR #3 OR #4 OR #5 OR #6 OR #7

#9AddSearch "Heparin"[Mesh:NoExp] OR "Unfractionated Heparin" OR "standard heparin" OR "standard heparine" OR "Unfractionated Heparine" OR "Conventional Heparin" OR "Conventional Heparine" OR "Heparinic Acid" OR Liquaemin OR "Sodium Heparin" OR "Sodium Heparine" OR "Heparin Sodium" OR "Heparine Sodium" OR "alpha-Heparin" OR "alpha-Heparine" OR "alpha Heparin" OR "alpha Heparine" OR Calciparin

#10AddSearch "Renal Dialysis"[Mesh:NoExp] OR "Renal Dialyses" OR "Renal Dialysis" OR Hemodialy* OR Haemodialy* OR "Extracorporeal Dialyses" OR "Extracorporeal Dialysis" OR "Extra-corporeal Dialyses" OR "Extra-corporeal Dialysis" OR "Extra corporeal Dialyses" OR "Extra corporeal Dialysis"
#11AddSearch #8 AND #9 AND #10

Literature search strategy for EMBASE and Cochrane central:

1	low molecular weight heparin/ or (LMWH or Low Molecular Weight Heparin or Low+Molecular+Weight Heparin).mp.
2	dalteparin/ or (Tedelparin or FR-860 or FR 860 or FR860 or Kabi-2165 or Kabi 2165 or Kabi2165 or Dalteparin Sodium or Fragmin or Pfizer Brand of Dalteparin Sodium or Fragmine or Dalteparine or Dalteparine sodique or Dalteparin sodique or Dalteparine sodium or Dalteparin natrium or Ligofragmin).mp.
3	enoxaparin/ or (Enoxaparine or Enoxaparin or Enoxaparin sodium or Enoxaparine sodium or Enoxaparin sodique or Enoxaparine sodique or PK-10,169 or PK 10,169 or PK10,169 or PK-10169 or PK 10169 or PK10169 or EMT-967 or EMT 967 or EMT967 or Lovenox or Clexane or EMT-966 or EMT 966 or EMT966 or Klexane).mp.
4	nadroparin/ or (Nadroparine or Nadroparin or Nadroparin Calcium or Nadroparine Calcium or Nadroparin Calcique or Nadroparine Calcique or Fraxiparin or Fraxiparine or CY 216 or CY-216 or CY216 or LMF CY-216 or LMF CY 216 or LMF CY216 or Fraxiparina).mp.
5	tinzaparin/ or (tinzaparin sodium or tinzaparine sodium or tinzaparin sodique or tinzaparine sodique or tinzaparin or tinzaparine or Innohep or Leo brand of tinzaparin sodium or Bristol-Myers Squibb brand of tinzaparin sodium or Logiparin).mp.
6	reviparin/ or (reviparine or reviparin or reviparine-sodium or reviparine sodium or reviparin-sodium or reviparin sodium or reviparine sodique or reviparin sodique or LU 47311 or LU-47311 or Clivarin or Abbott brand of reviparin-sodium or Clivarine or ICN brand of reviparin-sodium).mp.
7	bemiparin/ or (Bemiparin Sodium or Bemiparine sodium or Bemiparin sodique or Bemiparine sodique or Bemiparin or Bemiparine or Ibor or Zivor).mp.
8	1 or 2 or 3 or 4 or 5 or 6 or 7
9	heparin/ or (Unfractionated Heparin or standard heparin or standard heparine Unfractionated Heparine or Conventional Heparin or Conventional Heparine or Heparinic Acid or Liquaemin or Sodium Heparin or Sodium Heparine or Heparin Sodium or Heparine Sodium or alpha-Heparin or alpha-Heparine or alpha Heparin or alpha Heparine).mp.
10	renal replacement therapy/ or hemodialysis/ or (Renal Dialyses or Renal Dialysis or He?modialy* or Extracorporeal Dialys* or Extra+corporeal Dialys* or Extra corporeal Dialys*).mp.
11	8 and 9 and 10
12	limit 11 to (human and embase and Cochrane central)
