# Supplementary material for: Safety of low-molecular-weight heparin compared to unfractionated heparin in hemodialysis: a systematic review and meta-analysis
Source: BMC Nephrol. 2017 Jun 7;18:187. doi: 10.1186/s12882-017-0596-4 (PMC5463373; doi:10.1186/s12882-017-0596-4)
Supplement: Supplementary file 2 — Supplementary material providing the meta-analysis results for cholesterol (Table S1.) and triglycerides (Tables S2.), OPG/RANKL ratios (Table S3.), sensitivity analysis results (Table S4.) and funnel plots for publication bias. (PDF 177 kb) [file 12882_2017_596_MOESM2_ESM.pdf]

Table S1: Cholesterol weighted mean difference.

Study	LMWH	UFH	WMD (95% CI) (mg/dl)
	mean change, SD (mg/dl)	mean change, SD (mg/dl)	
Al-Saran et al**	-47.95 ± 57.87	-20.88 ± 38.81	-27.07 (-55.55, 1.41)
Deuber et al (part 1)	-44 ± 18.52	3 ± 29.72	-47.00 (-77.69, -16.31)
Deuber et al (part 2)	-73 ± 66.14	112 ± 49.03	-185.00 (-257.17, -112.83)
Elisaf et al	-21 ± 45.74	-5 ± 41.73	-16.00 (-35.68, 3.68)
Kronenberg et al	9.5 ± 43.13	-14.20 ± 42.05	23.70 (-0.40. 47.80)
Lai et al**	-7.34 ± 38.43	-14.69 ± 41.72	7.35 (-10.23, 24.93)
Leu et al**	-50.65 ± 43.15	25.13 ± 42.54	-75.78 (-102.34, -49.22)
Saltissi et al**	-1.16 ± 34.86	0 ± 35.58	-1.16 (-17.43, 15.11)
Schmitt et al	-30 ± 54.25	26 ± 52.03	-56.00 (-87.41, -24.59
Schrader et al	11 ± 66.78	5 ± 50.51	6.00 (-21.74, 33.74)
Summary			-28.70 (-51.43, -5.98)

**Results were expressed in mmol/L, we converted them in mg/dl to be able to pool them.

Table S2: Triglycerides weighted mean difference.

Study	LMWH<br>mean change, SD (mg/dl)	UFH<br>mean change, SD (mg/dl)	WMD (95% CI) (mg/dl)
Al-Saran et al**	-4.43 ± 168.40	-35.43 ± 72.30	31.00 (-43.90, 105.90)
Deuber et al (part 1)	-151 ± 31.48	-11 ± 83.22	-140.00 (-217.99, -62.01)
Deuber et al (part 2)	-120 ± 99.24	249 ± 128.21	-369.00 (-511.11, -226.89)
Elisaf et al	-15 ± 50.57	-1 ± 77	-14.00 (-43.29, 15.29)
Kronenberg et al	-0.20 ± 90.01	-27.3 ± 86.96	27.10 (-22.97, 77.17)
Lai et al**	-31.00 ± 63.62	-11.52 ± 108.19	-19.48 (-53.38, 19.42)
Leu et al**	-28.34 ± 285.14	18.60 ± 273.65	-46.94 (-220.15, 126.27)
Saltissi et al**	5.31 ± 53.60	-14.17 ± 71.13	19.48 (-9.61, 48.57)
Schmitt et al	-3 ± 105.70	38 ± 114.60	-41.00 (-106.14, 24.14)
Schrader et al**	0 ± 122.50	68 ± 156.70	-68.00 (-133.90, -2.10)
Stefoni et al	-28.90 ± 72.07	3.10 ± 74.50	-32.00 (-59.65, -4.35)
Yang et al (part 2)	-146.7 ± 89.76	64.30 ± 63.44	-211.00 (-279.12, -142.88)
Summary			-55.57 (-94.49, -16.66)

**Results were expressed in mmol/L, we converted them in mg/dl to be able to pool them.

Table S3: Osteoporosis expressed by OPG/RANKL ratio changes.

		OPG				RANKL			
		LMWH		UFH		LMWH		UFH	
Study	Measure type	Start of study	End of study	Start of study	End of study	Start of study	End of study	Start of study	End of study
Cianciolo et al	Mean ± SD (log transf)	2.66 ± 0.98	2.85 ± 0.96	2.96 ± 0.85	2.80 ± 091	1.07 ± 1.13	0.83 ± 0.60	2.17 ± 1.84	1.24 ± 1.0
Klejna et al	Median (range)	10.1 (4.2-28.1)	10.9 (4.6-26.5)	11.1 (4.9-19.1)	10.6 (5.7-33.4)	109 (40-301)	151.2 (43.4-386)	144 (83-361)	136 (81.7-315)

Table S4: Sensitivity analyses with for cholesterol, triglycerides, LSL-cholesterol with different correlation factors

**Outcome**	**Correlation factor of 0.3**	**Correlation factor of 0.5**	**Correlation factor of 0.8**
	WMD (%95 CI)	WMD (%95 CI)	WMD (%95 CI)
**Total cholesterol**	-27.38 (-50.78, -3.98)	-28.70 (-51.43, -5.98)	-32.25 (-55.08, -9.42)
**Triglycerides**	-53.76 (-94.98, -12.53)	-55.57 (-94.49, -16.66)	-60.78 (-97.74, -23.83)
**LDL-cholesterol**	-14.49 (-35.73, 6.75)	-14.88 (-36.27, 6.51)	-15.49 (-37.56, 6.57)

WMD: weighed mean difference in mg/dl; results from correlation factor of 0.5 are the reference (main analysis)

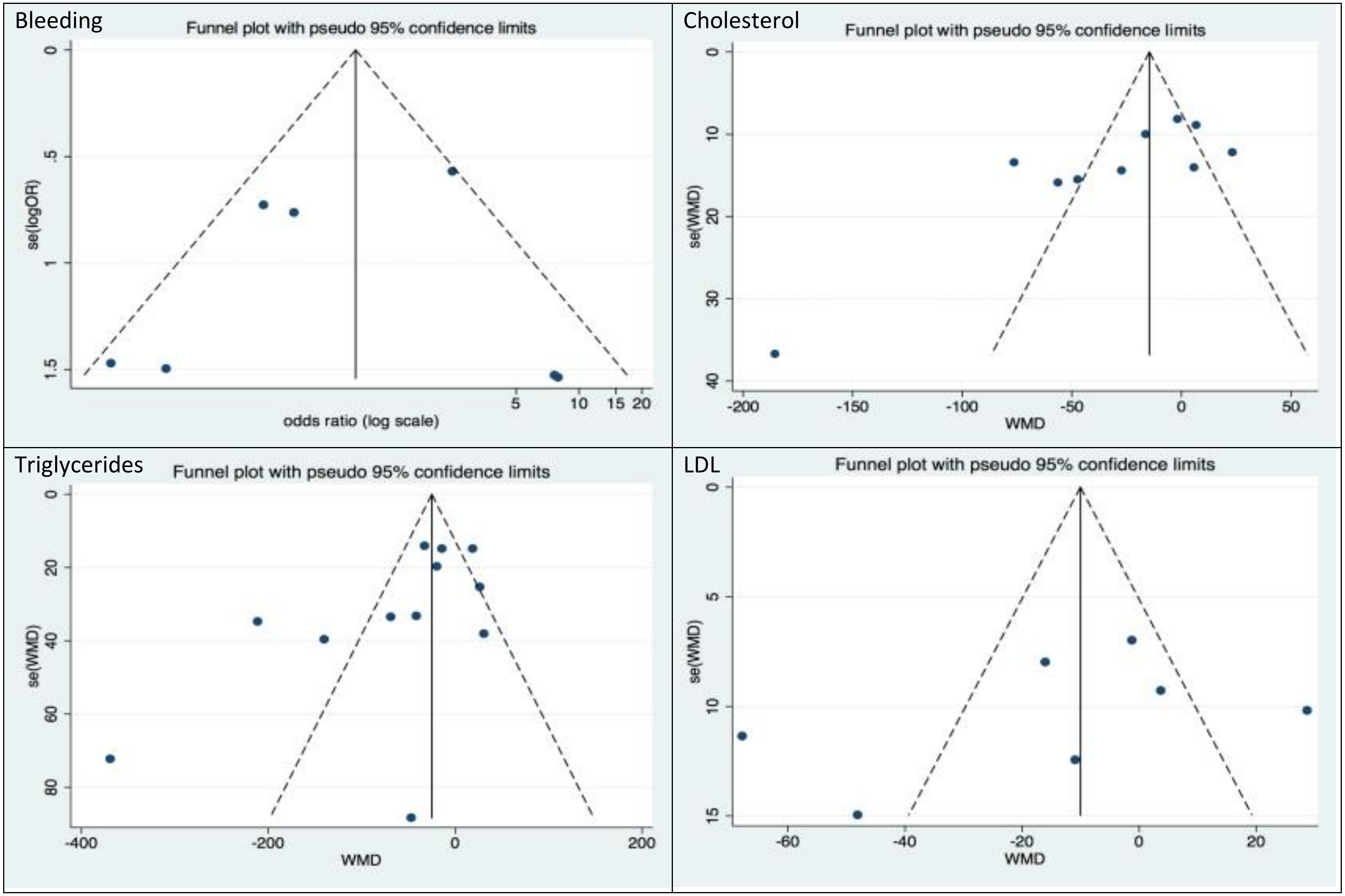

Supplementary figure S1: Funnel plots for publication bias evaluating bleeding, cholesterol, triglycerides and LDL-cholesterol
